# Long non-coding RNA *SeT* and *miR-155* regulate the *Tnfα* gene allelic expression profile

**DOI:** 10.1371/journal.pone.0184788

**Published:** 2017-09-14

**Authors:** Chrysoula Stathopoulou, Manouela Kapsetaki, Kalliopi Stratigi, Charalampos Spilianakis

**Affiliations:** 1 Department of Molecular Biology and Genetics, building 10 University Campus, Dragana Alexandroupolis, Greece; 2 Institute of Molecular Biology and Biotechnology-Foundation for Research and Technology Hellas, Nikolaou Plastira 100, Heraklion, Greece; 3 Department of Biology, University of Crete, Heraklion, Greece; University of South Alabama Mitchell Cancer Institute, UNITED STATES

## Abstract

It is becoming increasingly appreciated that the non-coding genome may have a great impact on the regulation of chromatin structure and gene expression. The innate immune response can be mediated upon lipopolysaccharide stimulation of macrophages which leads to immediate transcriptional activation of early responsive genes including tumor necrosis factor alpha (*Tnfα)*. The functional role of non-coding RNAs, such as lncRNAs and microRNAs, on the transcriptional activation of proinflammatory genes and the subsequent regulation of the innate immune response is still lacking mechanistic insights. In this study we wanted to unravel the functional role of the lncRNA *SeT*, which is encoded from the murine *Tnfα* gene locus, and *miR-155* on the transcriptional regulation of the *Tnfα* gene. We utilized genetically modified mice harboring either a deletion of the *SeT* promoter elements or the mature *miR-155* and studied the response of macrophages to lipopolysaccharide (LPS) stimulation. We found that decreased expression of the lncRNA *SeT* in murine primary macrophages resulted in increased mortality of mice challenged with LPS, which was corroborated by increased *Tnfα* steady state mRNA levels and a higher frequency of biallelically expressing macrophages. On the contrary, *miR-155* deletion resulted in reduced *Tnfα* mRNA levels supported by a lower frequency of biallelically expressing macrophages upon stimulation with LPS. In both cases, in the absence of either lncRNA *SeT* or *miR-155* we observed a deregulation of the *Tnfα* allele homologous pairing, previously shown to regulate the switch from mono- to bi-allelic gene expression. Although lncRNA *SeT* was not found to be a direct target of *miR-155* its stability was increased upon *miR-155* deletion. This study suggests a role of the non-coding genome in mediating *Tnfα* mRNA dosage control based on the regulation of homologous pairing of gene alleles and their subsequent biallelic expression.

## Introduction

Innate immunity constitutes the first defensive line against pathogen invasion [1]. Macrophages (Mφs), the main cellular component of innate immunity, can be activated by, among others, interleukins (IL-4, IL-10), interferon-γ (IFN-γ), or Gram-negative bacterial lipopolysaccharide (LPS), in order to exert their homeostatic role under normal, pathogenic and tumorigenic conditions [2–6]. LPS stimulation of Toll-like receptor 4 (TLR-4) initiates the pro-inflammatory nuclear factor κB (NF-κB) signaling pathway leading to both transcriptional and conformational changes of the chromatin landscape orchestrating inflammatory gene activation in the nucleus [7–9]. Effectors of the non-coding genome impact both on genomic architecture maintenance and on the transcriptional regulation of innate immune responses [10,11].

The long non-coding RNAs (lncRNAs) are RNA transcripts exceeding 200 nucleotides (nt) in size that can be traced either in the nucleus or the cytoplasm in a polyadenylated or not form [12,13]. Regarding their location relative to protein coding genes they can be further separated into intronic, long intergenic (lincRNAs), bidirectional, antisense and pseudogene lncRNAs [14]. Regarding their functional role, ncRNAs can interact with target RNA, DNA sequences or even proteins and are implicated in both gene silencing or activation or serve as a decoy mechanism competing with other regulatory proteins for binding in specific sites [15–17]. Other functional roles attributed to lncRNAs are the scaffold platform for other proteins or small RNAs, or the role of guiding proteins in specific sequences facilitating genomic interactions [18–21]. In the innate immune system specifically, the ~2 kb *THRIL* (linc1992) lncRNA was found significantly induced in stimulated human monocytes and was shown to upregulate tumor necrosis factor alpha (TNFα) pro-inflammatory cytokine transcription through a complex formation with hnRNPL on *Tnfα* promoter [22]. Additionally, the ~2,6 kb *NKILA* lncRNA was found to downregulate cancer-related inflammation pathways by binding to the p65 component of the NF-κB complex and masking the phosphorylation sites responsible for IκB-mediated release of the NF-κB complex in the nucleus [23].

Apart from lncRNAs, short non-coding RNAs, ~21 nucleotides long, termed microRNAs (miRNAs), are implicated in the initiation, progression and resolution of innate immune responses during both normal and pathological conditions [24,25]. Their functional role is summarized in silencing target-genes through sequence complementarity in the 3'-untranslated region (3'-UTR) of their mRNA targets and can be exerted either in the cytoplasm or the nucleus [26–28]. Among others *miR-146a*, *miR-let7e* and *miR-181c* target inflammatory components of the TLR pathway, exhibiting a suppressive role during innate immune responses [29–31]. On the contrary, *miR-155* supports the pro-inflammatory innate immune responses by enhancing *Tnfα* expression, or by facilitating IFN-I gene expression via suppression of cytokine signaling 1 (SOCS1) during the antiviral macrophage response [32,33]. The inflammatory role of *miR-155* is further accentuated by reports showing that *miR-155* deficient mice exhibit deregulated adaptive immune responses due to defective differentiation of B and T cells and antibody production, whereas their innate immunological profile downstream of LPS stimulation is characterized by decreased expression of pro-inflammatory cytokines such as IL-6 and TNF*α* [34,35].

Although inflammatory activation of macrophages is essential for pathogen eradication, flexible and temporally regulated skewing towards an anti-inflammatory cytokine profile is mandatory in order for host fitness to be preserved. Therefore, mRNA stability of inflammatory genes is modulated both at the transcriptional and post-transcriptional level by non-coding genome effectors (lncRNAs, miRNAs) and RNA binding proteins [AU-rich element (ARE)-binding proteins and RISC (RNA-induced silencing complex) components] [36–38]. At the post-transcriptional level, miRNAs along with (ARE)-binding proteins (TPP, TIA-1, TIAR or HuR) interact with the 3'-UTR of target mRNAs affecting their half-life. For instance, *miR-155* was shown to stabilize and enhance the cytoplasmic prevalence of *Tnfα* mRNA in both a heterologous system and cultured LPS-induced murine macrophages [32,39], whereas *miR-125b* and *miR-16* were shown to specifically target and destabilize *Tnfα* and *Il8* mRNAs respectively in an ARE-dependent manner [32,36,40]. At the transcriptional level, it is well-established that inflammatory gene transcriptional regulation is defined by signal transduction pathways culminating in NF-κB, AP-1 and IRF family members binding on regulatory enhancer and promoter sequences of primary and secondary response genes [41]. It is currently accepted that *in cis*-regulation of inflammatory genes can additionally be effectuated by lncRNAs transcribed in response to inflammatory stimuli by enhancers (elncRNAs) or promoters (plncRNAs) of the same inflammatory genes they regulate. For instance, the transcriptional activation of the two distal pro-inflammatory genes *PTGS-2(COX-2)* and *PLA2G4A* is regulated via *cis*-looping of their promoters [42,43]. Epigenetically regulated activation of *COX-2* is initiated by *miR-589* targeting of the *COX-2-*sense plncRNA resulting in RISC component sequestering to *COX-2* promoter [43]. The same gene is positively regulated by another extragenic, antisense transcribed lncRNA, *PACER*, which sequesters the NF-κB p65 subunit to induce inflammatory *COX-2* transcription [44].

*Tnfα* is a primary response pro-inflammatory gene, exerting a paracrine tumoricidal and an endocrine inflammatory role in macrophages of the innate immune system [45,46]. Except for macrophages, TNFα deficiency negatively affects both innate and adaptive inflammatory responses, impacting mainly on B cell follicle formation and increasing murine lethality after pathogenic infection [47,48]. On the contrary, prolonged TNFα production escorted by unresolved inflammation is linked to tissue damage and septic shock and is therefore counterbalanced by the collateral activation of immunosuppressive signaling pathways favoring macrophage tolerance after recurrent LPS stimulation events [49–51]. Thus, *Tnfα* mRNA production should be under dosage control and regulated both at the transcriptional and post-transcriptional level.

The *Tnfα* locus encompasses the tandemly arranged genes coding for TNFα, Lymphotoxin alpha (*Ltα*) and Lymphotoxin beta (*Ltβ*) and is mapped within the class III region of the major histocompatibility complex (MHC), occupying ~12 kilobases of genomic DNA on human chromosome 6 and mouse chromosome 17. Tight control of *Tnfα* gene expression in specific cell types and after specific stimuli is essential for cellular homeostasis and normal physiology. This is evidenced by the fact that deregulated TNFα levels are associated with multiple disease states. While TNFα deficiency has been linked to differential susceptibility to infections, resulting in complete lack of B cell follicles or causing tuberculosis, prolonged high concentrations of TNFα can result in severe tissue damage, asthma, rheumatoid arthritis, cardiovascular diseases, inflammatory bowel disease, type II diabetes, systemic lupus erythematosus, psoriasis, septic shock and cancer [52]. It is thus evident that a tightly regulated balance of TNFα levels is of critical importance. Transcriptional activation and expression of *Tnfα* downstream of the NF-κB signaling pathway is cell type and stimulus dependent and regulated in a spatiotemporal manner [53,54]. *Tnfα* monoallelic expression downstream of TLR4 activation in murine macrophages precedes the homologous association of the two alleles followed by biallelic *Tnfα* expression early upon the initiation of the inflammatory response [54].

We have previously shown that the 12.34 kb *SeT* lncRNA, which is encoded by the *Tnfα* gene locus, is significantly upregulated in LPS-induced murine macrophages and transcribed in a *Tnfα*-sense orientation. *SeT* lncRNA is involved in the regulation of the spatiotemporal expression of the *Tnfα* gene affecting its allelic expression profile [54]. Although crucial for the fine-tuning of immune responses, the mechanism by which the spatiotemporal regulation of *Tnfα* expression is effectuated, by means of homologous pairing and mono- to bi-allelic switch in transcription, is not fully elucidated. We thus sought to investigate the role of lncRNA *SeT* in this series of events and shed light on the regulatory functions of ncRNAs in the transcription profile of macrophages.

## Materials and methods

### Mice

For the conducted experiments mice were maintained at the Institute of Molecular Biology and Biotechnology (IMBB) colony and experiments were approved by the General Directorate of Veterinary Services, Region Crete (permit numbers: EL91BIO-02). The murine strains used were: C57BL/6, *miR-155*^*-/-*^ (B6.CgMir155<tm1.1Rsky>/J) obtained from the Jackson Laboratory, C56BL/6 meox2-Cre (B6.129S4-Meox2tm1(Cre)Sor/J) obtained from the Jackson Laboratory, C56BL/6 CX3CR1-Cre mice were kindly provided by Steffen Jung, CX3CR1-Cre *SeT*^*fl/fl*^ mice and *SeT* null mice (*SeT*^-/-^) were generated on C57BL/6 genetic background as follows: The targeting construct was built on a traditional targeting vector with TK–neo cDNA flanked by loxP sites. For the conditional knockout an extra loxP site was introduced in the short arm of the homologous sequence. The homologous sequences were produced by PCR with the Expand High Fidelity kit (ROCHE, Cat.No. 11732641001) and the specific primers used are listed in Table 1. The complete targeting construct was sequenced and thoroughly checked for any nucleotide substitutions and was introduced by electroporation in mouse embryonic stem cells of C57BL/6 genetic background, obtained by EUCOMM (C57BL/6N JM8A3 Agouti Black6 ES cells). For the preparation of the *SeT*^-/-^, the *SeT*^*fl/fl*^ mouse was crossed with a mouse of C56BL/6 meox2-Cre strain. Each genotype was determined by genomic PCR (Table 1).

**Table 1:** Primers used for the targeting construct preparation and genotyping.

	Primer #	Sequence 5’- 3’
**Homologous regions amplification for targeting construct preparation**		
Short arm	1074	GCC*ACTAGT*CAGGCAGAC*AGATCT*TATTGTTCTTCC
	1076	GCC*GCGGCCGC*CTGTCCTAGAATGTTCCAGGTCTG
Floxed region	1109-loxP	CCG*AGATCT*ATAACTTCGTATAGCATACATTATACGAAGTTATCTGTCTGCCTGAGAGAGAGC
	1075	GCC*ACTAGT*CTGGCTCAGGTAGAAGTTCCATC
Long arm	1077	GCC*AAGCTT*CAGAAATTAGAGCTGGACATTGTCC
	1078	GCC*GTCGAC*AGAATCCCTCGGAGACTGAAACC
**Genotyping primers**		
Common	506	ACCTGGGCCTTTTCTTCAG
Mutated-specific	576	GGGAAGGGCAATACTATTAGGT
Wild type-specific	770	CAGACGAAGGAAGGGTAAGC
Floxed-specific	1161	CGACTGCATCTGCGTGTTC

### *In vivo* LPS challenging and survival rates

Animals had an average weight of 22 g (range, 19–25 g) prior to the start of the experiments. Animal husbandry conditions included a room temperature of 23°C, humidity of 50%, and a 12-hour light–dark cycle (dark from 19:00 h to 07:00 h). Bedding in cages consisted of sawdust and wood shavings. Animals were housed with one to three cage mates. LPS (SIGMA, L2630) was diluted in pyrogen-free 1X PBS and injected to C57BL/6 wild type (WT) and *SeT*^-/-^ mice. For the experiments with an intraperitoneal injection of lethal LPS dose, twelve WT (9x male, 3x female) and twelve *SeT*^-/-^ (5x male, 7x female) mice, 8–12 weeks old, were intraperitoneally injected with a lethal LPS dose (500 μg LPS were injected in female mice weighing 19–25 gr and 600 μg LPS were used for male mice weighing 25–34 gr). One WT and one *SeT*^-/-^ mouse were injected with PBS only without LPS. Survival of animals was monitored for 3 days. For the experiments with an intraperitoneal injection of lethal LPS dose, 8–12 weeks old, twelve WT (C57BL/6, six males and six females) and twelve *SeT*^-/-^ (five males and seven females) mice were intraperitoneally injected with a sublethal dose of LPS (10 mg LPS/kg of body weight), their survival was monitored for 14 days and the survivors (seven WT and eight *SeT*^-/-^ mice) were re-challenged with a lethal dose of 25 mg/kg LPS/body weight. Control mice for both wild type and knockout counterparts were injected only with sterile 1X PBS. Survival of animals was monitored for 5 days.

Pain from the injection was assessed using facial expression as well as body posture and vocalization. Monitoring of the health of the animals was conducted by two investigators (the principal investigator and a veterinarian) every 1 hour after the LPS injection for 8 hours, and thereafter every 1 hour over a 12 hour period per subsequent day. Mice were evaluated while they were still in their cages (with the lids removed for better visualization). Certain variables such as temperature and weight loss did not change during the experimental timeline, while no mice needed analgesia for pain immediately after the LPS injection. The specific criteria used to determine when animals should be euthanized included LPS-related severe illness as indicated by reduced mobility, body posture, inability to eat and drink and a lack of response when gently prodded with forceps. Once animals reached endpoint criteria they were euthanized within the next ten minutes. There were no animals found dead before meeting the aforementioned criteria for euthanasia. All mice in the study were euthanized after they met the euthanasia criteria upon the LPS injection or after the conclusion of the study. The cause of death upon LPS injection was the induction of septic shock.

### Cell culture and treatments

All experiments were conducted according to institutional guidelines upon ethical committee approval. For peritoneal macrophage isolation from ten-week old C57BL/6, *SeT*^-/-^ and *miR-155*^-/-^ mice were intraperitoneally injected with 2 ml of 4% w/v thioglycollate medium (Brewer’s medium, LAB064) diluted in 1X Phosphate-Buffered Saline (PBS). Four days after the injection the mice were sacrificed and washed intraperitoneally with 15 ml filtered saline. Following the peritoneal lavage the cells were centrifuged and resuspended in fresh Dulbecco's Modified Eagle's Medium [DMEM, (GIBCO, Cat.No.41966)] supplemented with 10% fetal bovine serum (GIBCO, Cat.No.10270), 2 mM L-Glutamine (GIBCO, Cat.No.15030), 1X penicillin and streptomycin (GIBCO, Cat.No.15140122) under sterile conditions. The cells were seeded on culture dishes and incubated under 5% CO_2_ at 37°C for at least 24 hours before any experimental treatment.

Bone marrow derived cells were isolated from 10-week old C57BL/6, CX3CR1-Cre *SeT*^-/-^ and *miR-155*^-/-^ femurs and tibia as previously described [55]. Isolated cells were pelleted and resuspended in differentiation medium [Dulbecco's Modified Eagle's Medium supplemented with 10% fetal bovine serum, 2mM L-Glutamine, 30% custom-made L929 conditioned medium]. The cells were seeded in separate ventilated-cap flasks and incubated for 7 days under 5% CO_2_ at 37°C in order to differentiate. All murine primary macrophages were stimulated with 50 ng/mL LPS (EB Ultrapure, Invivogen, O111:B4) in various time frames depending on the experimental setup. LPS re-challenging experiments, in 1 hour re-stimulated peritoneal macrophages, were conducted as previously described [56].

### cDNA synthesis

For quantitative *Tnfα* and *SeT* RNA expression analysis in WT, *miR-155*^-/-^ and *SeT*^*-/-*^ murine macrophages, whole cell RNA was prepared using the TRI-REAGENT (SIGMA, T9424) following the manufacturer’s instructions. To eliminate genomic DNA contamination of the RNA samples, 5 μg of total RNA were treated with 10 units of DNase I (New England Biolabs, M0303L) for 1 hour at 37°C. The DNA-depleted RNA preparations were ethanol precipitated in the presence of 2 μg of linear acrylamide (Ambion, Cat.No 9520). After centrifugation for 20 min at 4°C, the RNA pellets were washed with 75% ethanol and reconstituted in DEPC-treated H_2_O. 500 ng of precipitated RNA and either 50 pmol of oligo-dT primer or 2 pmol of gene-specific primer were denatured for five minutes at 70°C. For each reverse transcription reaction, 200 units M-MuLV Reverse Transcriptase (NEB, M0253S) or Superscript III (Invitrogen, Cat.No.18080-044) were incubated with each RNA sample for two hours at 42°C or 50°C respectively. In parallel, control reactions deprived of the enzyme were prepared. 10% of the cDNA produced was used for each qPCR reaction, performed with the use of SYBR Select PCR Master mix (Applied Biosystems, Cat.No.4472908) according to the manufacturer’s instructions. The thermal cycler used for the aforementioned reactions was an Opticon 2 DNA Engine (MJ Research) creating standard curves. Reactions were performed in triplicates for statistical evaluation. The primer sets used for quantitation of *Tnfα* and lncRNA *SeT* primary transcripts are listed in Table 2.

**Table 2:** Primer pairs used for *Tnfα*, and *SeT* nascent RNA detection after whole cell RNA extraction and qPCR.

Gene name	Sequence 5’- 3’	Product size (bp)
PCR#1.F	CTGATGGTAGCCGAGACG	217
PCR#1.R	TCTCCATCATCCCCTTATGCACC	
PCR#2.F	GGGAAGGGCAATACTATTAGGT	175
PCR#2.R	GAATGAGTGACAGCCTAAGACG	
PCR#3.F	ACTGTGTCCCCTTACTCTCTG	315
PCR#3.R	CAGAGCATTGGAAGCCTGG	
PCR#4.F	GCTTGAGAGTTGGGAAGTGTG	130
PCR#4.R	AGGAGAGGCTTGTGAGGTC	
PCR#5.F	AGAGGGAGGCCATTTGGGAA	142
PCR#5.R	CTCCAGGCGGTGCCTATGT	
PCR#6.F	AGAGCCTTCCAGTGGGGTGAGA	92
PCR#6.R	ACAAGGAAGGCAATGACTAGG	
*Hprt1*.F	GTCCCAGCGTCGTGATTAGC	86
*Hprt1*.R	TTCCAAATCCTCGGCATAATG	

The absolute quantities of *SeT* cDNA as well as the *Tnfα* cDNA in *miR-155*^-/-^ and WT mice were calculated based on a standard curve, while the relative quantities of *Tnfα* RNA in WT versus *SeT*^-/-^ mice were calculated based on the –ΔΔC(t) method [57]. The values regarding the quantitative differences in qPCR triplicates were expressed as mean standard deviation (±SD). Data presentation graphs were designed using the SigmaPlot software.

### BAC clone culture

The murine BAC clone of the *Tnfα* locus used for the preparation of DNA-FISH probes was purchased in the form of bacterial glycerol stocks from BACPAC Resources Centre, CHORI. The aforementioned clone was: *Tnfα* locus (chr.17): RP23-446C22. The BAC clone was grown on custom made Luria-Bertani (LB) Broth supplemented with chloramphenicol in a final concentration of 12.5 μg/ml. BAC DNA was isolated after bacterial cell lysis, RNase-treated, phenol-chloroform extracted and ethanol precipitated. Reconstituted BAC DNA concentration and purity were estimated with both electrophoretic and spectrophotometric assays. BAC clone confirmation was performed with conventional PCR reactions, using the following primer-set: BAC.*Tnfα*.F: 5’-GAAGAGCGTGGTGGCCC-3’, BAC.*Tnfα*.R: 5’-CTCCAGGCGGTGCCTATGT-3’.

### Probe construction for RNA-DNA FISH and *SeT* riboprobe preparation

The DNA FISH probe for the *Tnfα* locus (chr.17): RP23-446C22 was constructed using the Vysis Nick Translation kit (Abbott Molecular, 07J00-001) supplemented with Spectrum Orange dUTP (Abbott Molecular, 02N33-050/02N32-050). Each reaction was prepared with 3 μg BAC DNA, following the kit’s manual. Each probe was purified using the Purelink PCR purification kit (Invitrogen, K31001). *Tnfα* cDNA FISH probe was constructed using the Vysis Nick Translation kit supplemented with Spectrum Green dUTP based on its cDNA sequence cloned to a pCR® 2.1 plasmid vector using the *Tnfα*.F: 5’-ATGAGCACAGAAAGCATGATCCG-3’, *Tnfα*.R: 5’-TCACAGAGCAATGACTCCAAAGT-3’ primer-set. Strand-specific RNA probes for lncRNA *SeT* detection were prepared with *in vitro* transcription of PCR products obtained with the *SeT*.F: 5’-GGCCTAATACGACTCACTATAGGGAGAACTGGCCATGGGACCCAC-3’, and *SeT*.R: 5’-TCTCCATCATCCCCTTATGCACC’3’ primer-set, yielding a 379 bp PCR product, containing the T7 promoter on the 5’-end. The *SeT* lncRNA riboprobe was prepared with the Biotin NT labeling kit (Jena Biosciences, Cat.No PP-310-BIO16) and precipitated with LiCl.

### RNA fluorescence *in situ* hybridization (RNA FISH)

Cell preparation for RNA FISH of C57BL/6, *SeT*^*-/-*^ and *miR-155*^*-/-*^ bone-marrow-derived macrophages entailed their seeding on sterile glass coverslips, and LPS stimulation. Cells were then transferred on ice and washed once with ice cold 1X PBS. Subsequently, they were fixed in 4% PFA/1X PBS for 10 minutes, permeabilized with 0.5% Triton X-100/1X PBS for 5 minutes and rinsed repeatedly with 1X PBS. Before hybridization cells were dehydrated in 70% ethanol.

In order to prepare the cells for RNA FISH hybridization, macrophages were dehydrated with ethanol washes of increasing concentration (70%, 80%, 95% and 100%) and briefly air-dried. Each probe preparation was consisted of 30 ng biotin-labeled *SeT* riboprobe, along with 20 μg yeast transfer RNA (Ambion, Cat.No AM 7119), lyophilized and resuspended in 5 μl de-ionized formamide. After reconstitution, probes were mixed thoroughly with 5 μl of 2X hybridization buffer [4X SSC, 20% Dextran sulfate, 2 mg/ml acetylated BSA (Ambion, Cat.No AM 2614-G1, 50 mM Sodium Phosphate)] and placed on a glass microscope slide. The cell-spotted side of the coverslip was flipped on top, sealed with rubber cement and incubated for 16 hours in a humidified hybridization chamber at 37°C.

### RNA-DNA fluorescence *in situ* hybridization (RNA-DNA FISH)

Cell preparation for RNA-DNA FISH required seeding and LPS-stimulation of macrophages (either bone marrow- or peritoneally-derived), which were then transferred on ice and washed with ice cold 1X PBS. For cytoplasm removal, cells were treated for 3 minutes with cytoskeletal buffer (CSK) containing 100 mM NaCl, 300 mM sucrose, 3 mM MgCl_2_, 10 mM PIPES, 0.5% Triton X-100, 1 mM EGTA, and 2 mM vanadyl-ribonucleoside complex (NEB, Cat.No S1402S). Cells were then fixed for 10 minutes with 4% PFA in 1X PBS, dehydrated twice with 70% ethanol for 3 minutes and stored in 70% ethanol at -20°C until use.

For RNA-DNA FISH cell preparation for hybridization, the procedure followed was similar to the one previously described for RNA FISH. 100 ng from each *Tnfα* DNA or cDNA probe, and 30 ng of indirectly labeled *SeT* riboprobe, along with 1 μg mouse COT-1 DNA (Invitrogen, Cat.No.18440-016) and 20 μg yeast transfer RNA were lyophilized and resuspended in 5 μl de-ionized formamide. After reconstitution, probes were denatured at 95°C for 10 minutes and then mixed thoroughly with 5 μl of 2X hybridization buffer (4X SSC, 20% Dextran sulfate, 2 mg/ml acetylated BSA). The hybridization mix was placed and sealed with the cell-spotted side of the coverslip and incubated for 16 hours in a humidified hybridization chamber at 37°C.

Following hybridization, preparations with directly labeled probes were mildly washed with 2X SSC at room temperature, coverslips were then dried and nuclear DNA was counterstained with DAPI. Preparations with biotin-labelled *SeT* RNA were processed using the TSA Biotin System (Perkin Elmer, Cat.No NEL700A001K) according to the manufacturer’s instructions. Briefly, cell-spotted coverslips were rinsed three times with each of the following buffers: 2X SSC in 50% formamide, 2X, 1X, 0.5X, 0.2X and 0.1X SSC buffer, for 10 minutes. The cells were then blocked for 30 minutes with TNB buffer (100 mM Tris-HCl pH 7.5, 150 mM NaCl, 0.5% blocking reagent provided with the kit) in a dark humid chamber and then incubated with streptavidin (SA) conjugated with horseradish peroxidase (HRP) in a dilution of 1/200 in TNB buffer for additional 30 min at room temperature. The hybridized cells were washed twice with TNT buffer (100 mM Tris-HCl pH 7.5, 150 mM NaCl, 0.05% Tween 20) for 5 minutes at room temperature and then incubated for 5 minutes at room temperature with biotinylated tyramide in a dilution of 1/50 in amplification diluent (supplied by the kit) and rinsed twice with TNT buffer for additional 5 minutes. For visualization of the amplified RNA signal, cell-spotted coverslips were incubated with fluorophore-conjugated Streptavidin-488 (1/400 in TNB) for 30 minutes at room temperature, washed twice with TNT buffer and once with 1X PBS for 3 minutes each. The cells were subsequently dried and mounted with DAPI as previously described.

### Confocal microscopy and image analysis

RNA and RNA-DNA FISH signals were captured in stacks by a CCD camera of a high resolution Leica TCS SP8 confocal microscope. Images were scanned using a 63 oil objective lens and a z-axis step of 200 nm. The 3D deconvolution of the scanned images and the merge of sequential stacks were performed with the use of Volocity 3D Image Analysis Software (Perkin Elmer).

### MicroRNA target prediction

The prediction of *miR-155* target sequences on the *Tnfα* locus was performed using the microRNA target prediction tool *Targetprofiler*, a hidden Markov type model trained on experimentally verified miRNA targets, as developed and described by A.Oulas et al. [58].

## Results

### *SeT* lncRNA deletion increases *Tnfα* mRNA levels

**Fig 1:**
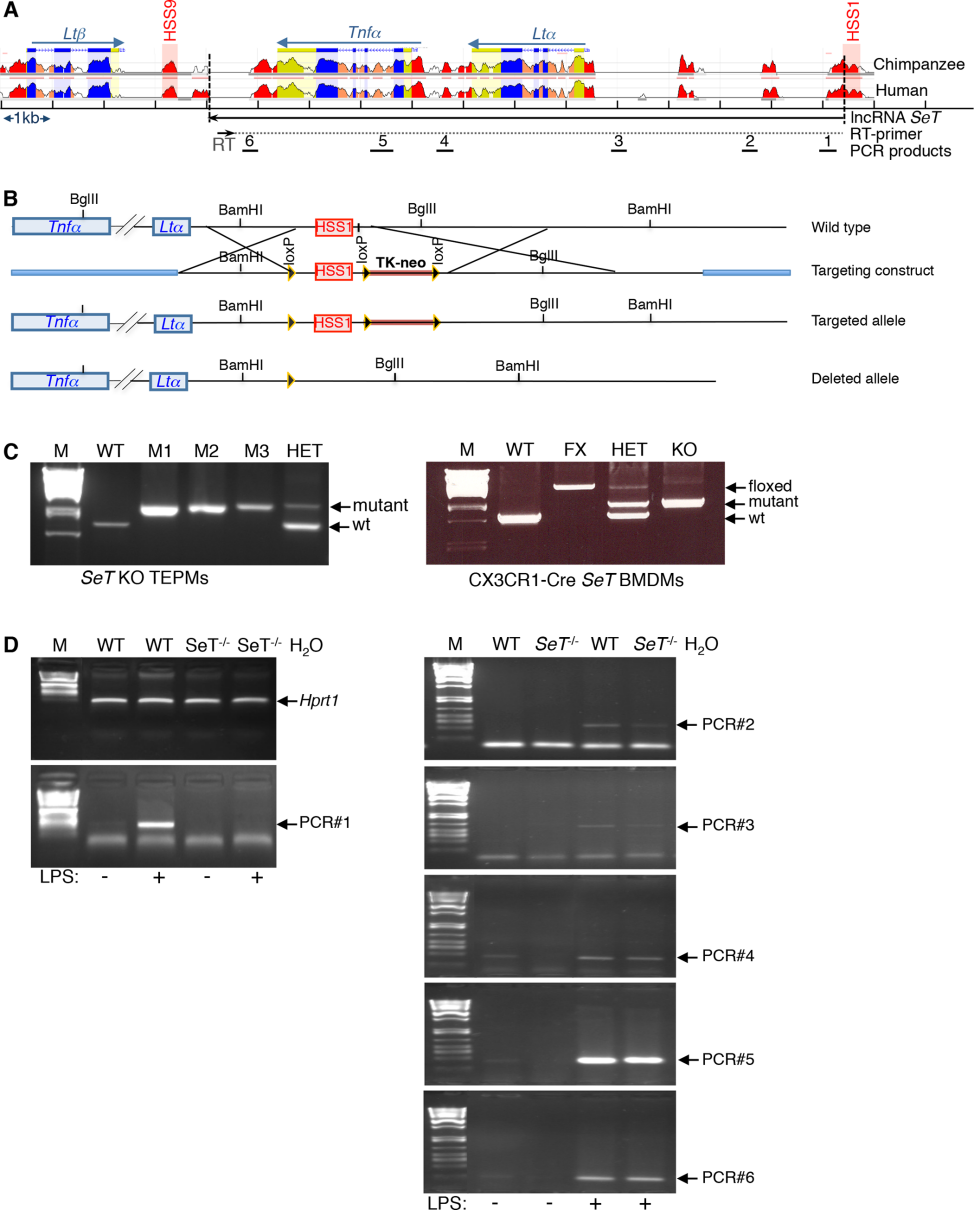
*SeT* deletion in primary murine macrophages. (A) Representative graph of the murine *Tnfα* gene locus indicating the homology (>70%) with the human and chimpanzee genomes, mapping of the *SeT* lncRNA as well as the primer utilized for generating the *SeT* cDNA (RT) and the subsequent PCR products (1–6) with the *SeT* cDNA as template (blue: gene exons, yellow: untranslated regions, red: intergenic regions, arrows above genes: direction of gene transcription). (B) Diagrammatic representation of the targeting construct used for the *SeT*^*-/-*^ mouse generation. (C) Genotyping of the mutated *SeT* mice. Detection by RT-PCR of the deletion of 1647 bp region in genomic extracts of primary macrophages isolated from the peritoneum (TEPMs) or the bone marrow (BMDMs) of CX3CR1-Cre *SeT*^*fl/fl*^ mice and *SeT*^*-/-*^ BMDMs. M: MW marker, WT: wild type, M1-M3/KO: different genomic DNA samples derived from mice bearing the deleted allele in homozygosity, HET: heterozygote sample (bearing one wild type and one deleted allele for *SeT*), FX: floxed alleles. D) RT-PCR reactions for the detection of the relative mRNA levels for the lncRNA *SeT* and *Hprt1* gene transcripts (mapping of PCR products is indicated in panel A).

The *Tnfα* gene locus is located on mouse chromosome 17 and encompasses three genes involved in the regulation of the immune system, namely the lymphotoxin alpha gene (*Ltα*), the Tumor necrosis factor alpha gene (*Tnfα*) and the lymphotoxin beta gene (*Ltβ*) (**Fig 1A**). We have previously shown that the long non-coding RNA *SeT* is also expressed from the *Tnfα* locus and has an impact on the regulation of the homologous pairing of *Tnfα* alleles and ultimately the regulation of the allelic expression profile of the *Tnfα* gene [54]. In this study, we have targeted the *Tnfα* locus (**Fig 1B**) and created two mice with either constitutive deletion of the regulatory elements of the *SeT* lncRNA or tissue specific deletion of the same sequences in bone marrow derived macrophages (**Fig 1C**). We performed single primer reverse transcription of the lncRNA *SeT* and subsequently analyzed its expression by endpoint PCR (RT-PCR). Our analysis showed that deletion of the DNAse I hypersensitive region at the 5’-end of the lncRNA *SeT* resulted in the reduction of the *SeT* RNA levels as deduced by RT-PCR analysis performed for the whole transcript sequence in macrophages, before and after stimulation with lipopolysaccharide (LPS) (**Fig 1D**).

**Fig 2:**
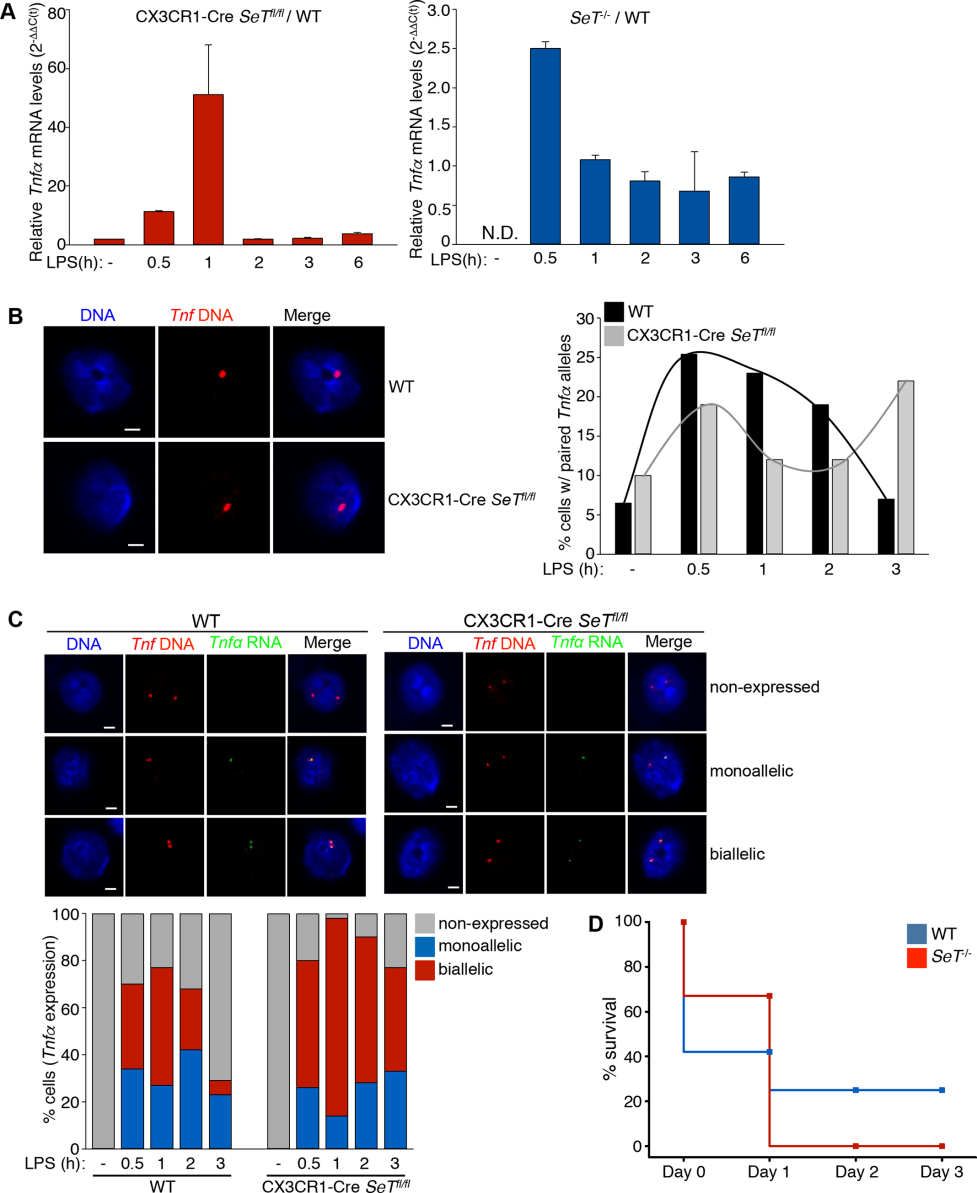
*Tnfα* expression in wild type and *SeT* deficient mice. (A) Relative *Tnfα* mRNA expression levels, calculated by the 2^-ΔΔC(t)^ method, showing overexpression of *Tnfα* in *SeT* deficient compared to wild type murine macrophages (BMDMs) upon LPS stimulation in a time frame of six hours. (B) Confocal-derived Z-sections portraying the homologous pairing of *Tnfα* alleles in wild type and CX3CR1-Cre *SeT*^*fl/fl*^ BMDMs, one hour upon LPS stimulation (scale bar 2 μm). Bar graph depicting the deregulated pattern of paired alleles between wild type and CX3CR1-Cre *SeT*^*fl/fl*^ BMDMs upon LPS stimulation for three hours. Only partially or completely overlapping DNA FISH signals were calculated as paired alleles (sample size, WT: 552 nuclei, CX3CR1-Cre *SeT*^*fl/fl*^: 495 nuclei). (C) Single-stack confocal images showing the frequency of mono- versus bi-allelic expression of *Tnfα* in wild type and CX3CR1-Cre *SeT*^*fl/fl*^ BMDMs (scale bar 2 μm) (sample size, WT: 552 nuclei, CX3CR1-Cre *SeT*^*fl/fl*^: 495 nuclei). (D) Survival rates of wild type and *SeT*^*-/-*^ mice expressed in percentage of mice out of the total number of individuals per genotype (12 mice per genotype) responding to the intraperitoneal injection of lethal LPS dose.

We then questioned whether this reduction in the *SeT* mRNA levels would have an impact in the *Tnfα* mRNA levels expressed in macrophages upon their induction with LPS. We performed quantitative RT-PCR using cDNA derived from isolated mRNA from bone marrow derived macrophages (BMDMs) of either wild type or knockout origin. Our analysis showed that the *Tnfα* mRNA levels expressed in BMDMs, upon their stimulation with LPS, from the knockout mice compared to their wild type counterparts were clearly increased (**Fig 2A**) in both the CX3CR1-Cre *SeT*^*fl/fl*^ and the *SeT*^*-/-*^ mice.

We have previously shown that *Tnfα* mRNA levels and more importantly the *Tnfα* allelic switch in expression from monoallelic to biallelic follows the homologous pairing of the two *Tnfα* gene alleles. Therefore, in order to test whether the deletion of *SeT* lncRNA had an impact on the *Tnfα* homologous pairing, we performed DNA fluorescence *in situ* hybridization for the *Tnfα* locus in CSK-treated (cytoskeletal buffer) BMDMs derived from either wild type or mutated mice. After quantitative analysis of the frequency of cell nuclei bearing *Tnfα* homologous pairing we found an altered pattern in *SeT* knockout BMDMs and a quite remarkable higher frequency of homologous pairing of *Tnfα* alleles three hours upon LPS stimulation (**Fig 2B**).

The *Tnfα* homologous pairing precedes and regulates the allelic expression profile of the *Tnfα* gene. Based on the deregulated pattern of *Tnfα* homologous pairing in the *SeT* knockout mice we questioned whether the allelic expression profile of the *Tnfα* gene was altered. To answer this we performed RNA-DNA FISH experiments for the *Tnfα* gene locus DNA and the *Tnfα* newly transcribed *Tnfα* mRNA in BMDMs derived from wild type and CX3CR1-Cre *SeT* ^*-/-*^ mice. We found that in the absence of the *SeT* transcript, there was a higher frequency of macrophages expressing the *Tnfα* gene, but more importantly the frequency of cells expressing the *Tnfα* gene in a biallelic manner was greatly increased (**Fig 2C**).

To examine whether the increased *Tnfα* mRNA levels detected in the knockout macrophages upon their stimulation with LPS, as deduced by the RNA-DNA FISH and qRT-PCR experiments, had an impact in the regulation of the innate immune system *in vivo* we challenged both wild type and *SeT* knockout mice with lethal doses of LPS and observed their survival in a time frame of three days after the challenge. Indeed, after forty-eight hours of LPS challenge all *SeT* knockout mice were deceased, while 25% of the wild type mice were still healthy and alive (**Fig 2D**).

In conclusion, we observed that the reduced lncRNA *SeT* mRNA levels had an impact in the *Tnfα* gene locus homologous pairing and ultimately the *Tnfα* allelic expression profile, resulting in higher *Tnfα* mRNA levels.

### *SeT* knockout macrophages become tolerant upon repeated LPS challenges

**Fig 3:**
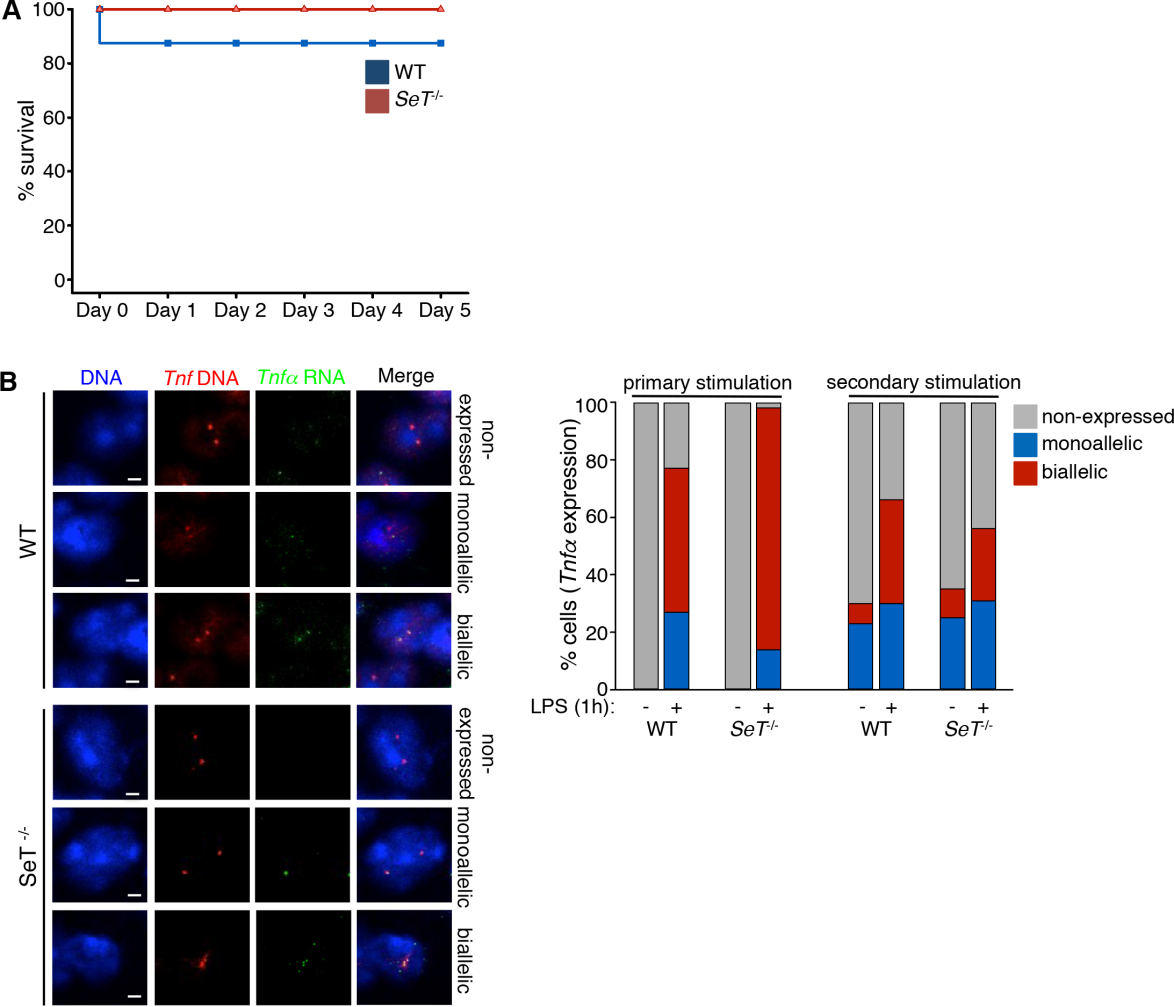
*Tnfα* allelic expression profile in tolerant wild type and *SeT*^-/-^ TEPMs. (A) Survival curves of wild type and *SeT*^*-/-*^ mice upon sublethal LPS dose intraperitoneal injection and re-challenging with a lethal LPS dose. The survival rate of each germline is defined by the percentage of surviving individuals over the total number of mice per genotype. (B) Single-stack confocal images portraying the mono-/bi-allelic expression profile of *Tnfα* in tolerant and one hour LPS-restimulated TEPMs (scale bar 2 μm). The graph represents the frequency of alleles expressed in either a mono- or a bi-allelic manner (sample size, WT: 646 nuclei, *SeT*^*-/-*^: 890 nuclei).

We have shown that the challenge of mice with lethal doses of LPS resulted in increased mortality of *SeT*^*-/-*^ mice supported by the fact that mutated macrophages express higher levels of *Tnfα* mRNA. We wanted to investigate whether repeated LPS challenges, known to reduce innate sensitivity to pro-inflammatory pathway activation, would differentially affect the innate immune response of wild type compared to *SeT* knockout mice, as previously reported for other mediators of the pro-inflammatory pathway [56,59]. Therefore, we performed *in vivo* challenging of both wild type and *SeT*^*-/-*^ mice with sublethal doses of LPS and after recovery, the surviving mice were re-challenged with a lethal LPS dose. We observed that upon the repeated challenge of mice with LPS there was no difference in survival of the wild type and *SeT*^*-/-*^ mice (**Fig 3A**). Actually, the fact that all mice showed negligible mortality suggested that *Tnfα* overexpression is somehow counterbalanced after recurrent LPS stimulations in the *SeT*^*-/-*^ mice, following the wild type pattern.

We therefore performed RNA-DNA FISH experiments in murine wild type and *SeT*^*-/-*^ macrophages and investigated the *Tnfα* expression levels of nascent transcripts before and after induced LPS tolerance of TEPMs. We measured the percentage of cells expressing *Tnfα* in an either a mono- or biallelic manner after both a primary and a secondary LPS challenge of mouse macrophages and found that the frequency of macrophages expressing the *Tnfα* gene was reduced upon the secondary LPS stimulation of all cells, irrespective of the *SeT* deletion. Moreover, the increased biallelic expression of the *Tnfα* gene in the *SeT*^*-/-*^ macrophages upon the initial LPS stimulation of the cells was not observed after the secondary LPS stimulation (**Fig 3B**). These data suggest that both wild type and *SeT*^*-/-*^ macrophages become tolerant upon re-stimulation with sublethal doses of LPS and control at the transcriptional level their *Tnfα* gene expression by reducing both the frequency of total expressing cells as well as the frequency of biallelically expressing cells.

### MicroRNA-155 regulates the allelic expression of the *Tnfα* gene independently of the *SeT* lncRNA

**Fig 4:**
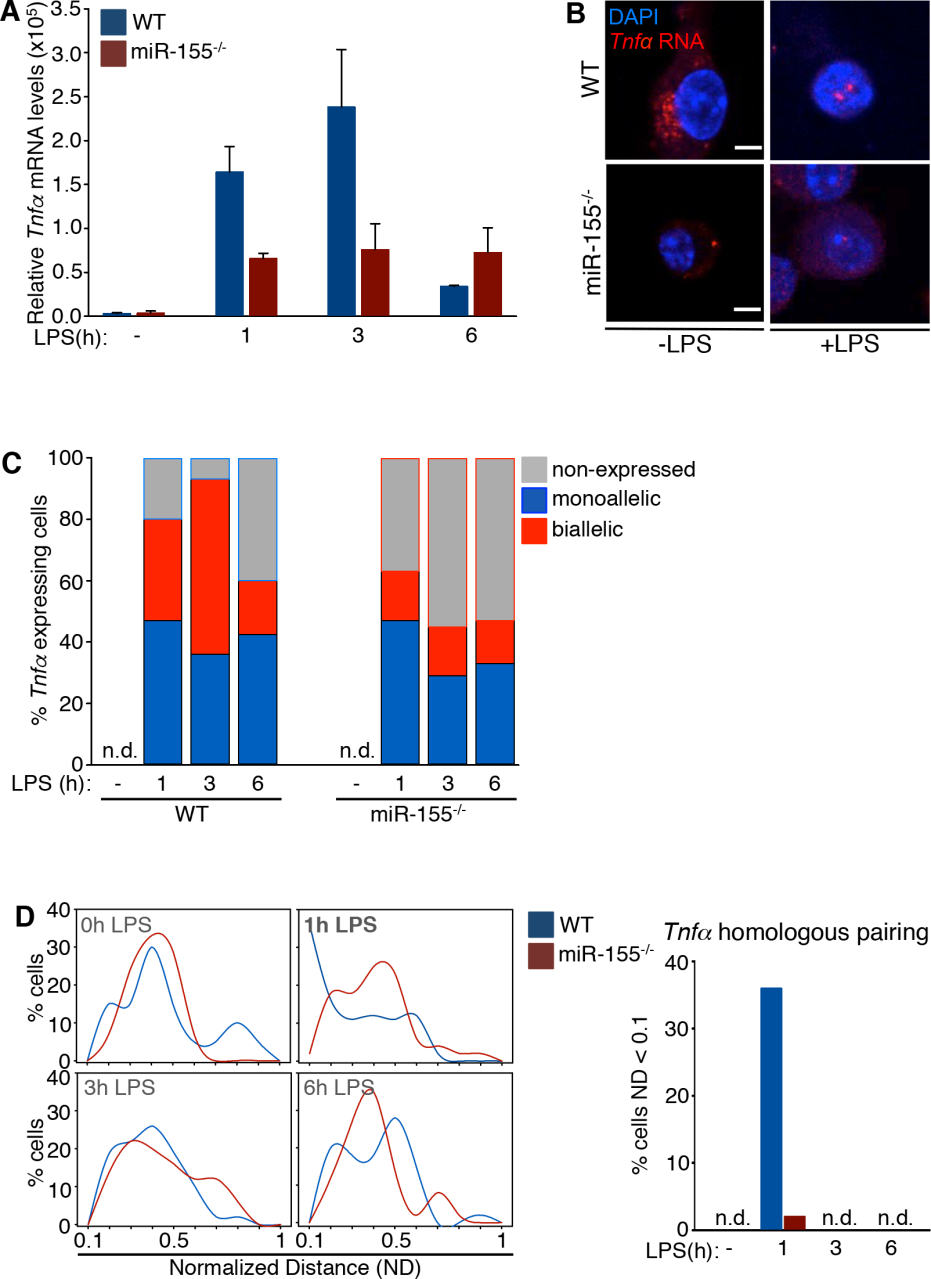
*Tnfα* mRNA expression in wild type and *miR-155*^*-/-*^ murine macrophages. (A) *Hprt1-*normalized relative mRNA expression levels of *Tnfα* analyzed by qRT-PCR in murine TEPMs isolated from either wild type or *miR-155*^*-/-*^ mice. (B) RNA FISH single-stack confocal images portraying the reduced nuclear and cytoplasmic *Tnfα* RNA in *miR-155*^*-/-*^ BMDMs, compared to wild type cells (scale bar 4 μm). (C) RNA-DNA FISH experiments for the *Tnfα* allelic expression profile in wild type and *miR-155*^*-/-*^ TEPMs (sample size, WT: 183 nuclei, *miR-155*^*-/-*^ 191 nuclei). (D) The *Tnfα* alleles distance was normalized for the volume of each cell (ND) and the frequencies of cells with a normalized distance from 0 to 1 were plotted (scale bar, 2 μm). For the far right graph, the frequency of cells with an allele ND < 0.1 was plotted (sample size, WT: 183 nuclei, *miR-155*^*-/-*^ 191 nuclei). (n.d.: not detected)

So far, our results indicated that the absence of *SeT* RNA impacts on *Tnfα* gene expression downstream of TLR4 activation at the transcriptional level. A recurrent LPS activation was found counterbalanced in a systemic level, impeding *Tnfα* over-expression, linked to increased mouse mortality due to septic shock. *MiR-155* is a master regulator of the pro-inflammatory innate immune response during primarily orchestrated innate responses, but also during macrophage tolerance, impacting on *Tnfα* gene transcription [35,59]. In order to elucidate to which extent the non-coding genome interactions are implicated in the regulation of *Tnfα* mRNA expression we initially assessed the impact of *miR-155* absence on the *Tnfα* steady state mRNA levels in murine macrophages. To study this we performed qRT-PCR experiments in wild type and *miR-155*^*-/-*^ mouse primary LPS-stimulated thioglycollate-elicited peritoneal macrophages within a time frame of six hours (**Fig 4A**). We found that the *Tnfα* steady state mRNA levels were reduced in the *miR-155*^*-/-*^ macrophages compared to their wild type counterparts. Our subsequent RNA FISH experiments, performed in both wild type and *miR-155*^*-/-*^ mouse macrophages, before and after LPS stimulation, revealed that the frequency of cells expressing *Tnfα* was greatly reduced in the *miR-155*^*-/-*^ cells (**Fig 4B**), both in the nuclear and the cytoplasmic compartment, further supporting our qRT-PCR analysis.

We then examined whether the overall reduction of nascent *Tnfα* transcripts was attributed to alterations of *Tnfα* allelic expression profile, plausibly due to the deregulated homologous association of *Tnfα* alleles between wild type and *miR-155*^*-/-*^ macrophages. The quantitation of the *Tnfα* allelic expression profile in the RNA-DNA FISH experiments, visualizing the *in situ* transcribed *Tnfα* nascent RNA showed that the detected frequency of nuclei expressing the *Tnfα* gene upon LPS stimulation in *miR-155*^*-/-*^ murine macrophages was lower compared to wild type cells (**Fig 4C**). These results are consistent with both our qRT-PCR and RNA FISH results and accentuate the link between sustained biallelic expression and increased total *Tnfα* mRNA production. As previously discussed, the *Tnfα* biallelic pattern of expression follows the homologous pairing of the two *Tnfα* alleles in mouse LPS-stimulated macrophages. We therefore tested the precedence of *Tnfα* alleles homologous pairing, prior the peak of *Tnfα* gene transcription, by measuring the distance of the two *Tnfα* alleles in *miR-155*^*-/-*^ mouse macrophages and their wild type counterparts. We found that in the *miR-155*^*-/-*^ cells the frequency of homologous paired *Tnfα* alleles was greatly reduced compared to the wild type macrophages, one hour upon LPS stimulation, which is the time point that precedes the maximal *Tnfα* expression levels at three hours upon LPS stimulation (**Fig 4D**). We conclude that in the absence of *miR-155* in mouse macrophages, the *Tnfα* gene expression levels are reduced upon LPS stimulation, the two *Tnfα* gene alleles do not pair and this results in the reduction of frequency of cell nuclei expressing the *Tnfα* gene in a biallelic manner.

**Fig 5:**
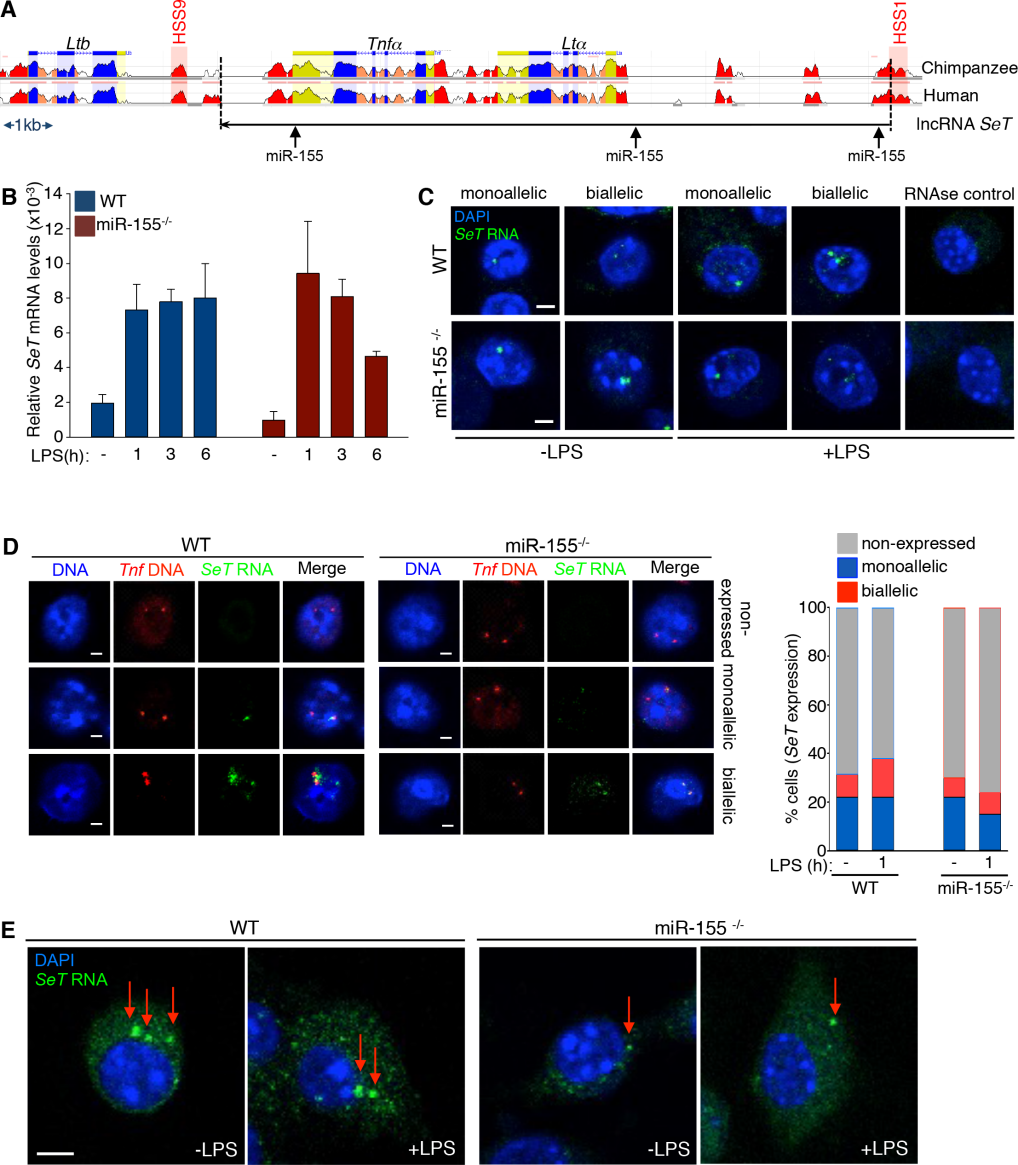
*SeT* RNA expression in wild type and *miR-155*^*-/-*^ BMDMs. (A) Depiction of three putative *miR-155* sequence complementarity seed sites on *SeT* RNA, as defined by the *Targetprofiler* prediction tool. (B) qRT-PCR analysis for the relative *SeT* RNA expression normalized with *Hprt1* mRNA levels in wild type and *miR-155*^*-/-*^ mice. (C) Detection of nascent *SeT* RNA under non-denatured conditions in conventional RNA FISH z-stack images before and after LPS induction of wild type and *miR-155*^*-/-*^ macrophages (scale bar 4 μm). (D) Single confocal z-stack RNA-DNA FISH images portraying the nascent mono- and biallelic expression of *SeT* RNA in naïve and one hour LPS-stimulated BMDMs of wild type and *miR-155*^*-/-*^ mice (scale bar 2 μm). The corresponding graph showing the percentage of total cells expressing *SeT* RNA in either a mono- or bi-allelic manner was based on measurements performed in two independent experiments (sample size, WT: 508 nuclei, *miR-155*^*-/-*^: 414 nuclei). (E) RNA FISH performed in naïve and LPS-stimulated BMDMs depicting the cytoplasmic detection of *SeT* RNA in wild type and *miR-155*^*-/-*^ BMDMs with the use of *SeT* biotinylated riboprobes under denatured conditions (scale bar 4 μm). Arrows indicate enlarged speckles of accumulated RNA in the cytoplasm.

LncRNAs are known interaction platforms for miRNAs, in some cases acting as sponges for the latter and in other cases being targeted and degraded by them and thus affecting coding-gene expression in both the cytoplasm and the nucleus [60,61]. We showed that both *miR-155* and *SeT* ablation can affect *Tnfα* expression via deregulation of homologous pairing of the two *Tnfα* alleles. We therefore searched for a possible link between *miR-155*/*SeT* RNA interaction and *Tnfα* transcriptional regulation in LPS-induced macrophages. By initially performing an *in silico* analysis we detected three putative *miR-155* seed sites on the *SeT* lncRNA sequence (**Fig 5A**). Next, we investigated the hypothesis of *miR-155* targeting the *SeT* lncRNA as a potential mechanism regulating the proinflammatory cytokine gene *Tnfα*. To test whether *SeT* lncRNA was a target of *miR-155* we stimulated macrophages from either wild type or *miR-155*^*-/-*^ mice and we performed qRT-PCR for the *SeT* transcript (**Fig 5B**). We found that the absence of *miR-155* had no major effect on the nascent steady state mRNA levels of the *SeT* transcript. To further support this finding we performed RNA FISH experiments for *SeT* lncRNA in naive and LPS-stimulated primary macrophages. Visualization of the nascent *SeT* RNA expression revealed a speckled nuclear pattern reminiscent of the mono- and bi-allelic pattern of *Tnfα* mRNA, which was though of lower intensity in the *miR-155*^*-/-*^ BMDMs as compared to wild type macrophages (**Fig 5C**). In order to perform quantitative measurements regarding the reduction of *SeT* RNA expression in murine macrophages, we performed RNA-DNA FISH experiments and detected both the *Tnfα* locus DNA and the nascent *SeT* RNA transcript in both wild type and *miR-155*^*-/-*^ mouse macrophages stimulated with LPS. We only assessed cell nuclei with one or two *SeT* RNA signals colocalized with the *Tnfα* locus and found that in the *miR-155*^*-/-*^ cells the allelic expression pattern for the lncRNA *SeT* was similar to the one observed in wild type macrophages (**Fig 5D**). By performing RNA FISH experiments on wild type and *miR-155*^*-/-*^ mouse macrophages and denaturing the cells before their hybridization with the biotinylated, strand-specific *SeT* RNA probe, we were able to efficiently detect the cytoplasmic *SeT* lncRNA transcript, without losing its corresponding nuclear signal. Surprisingly, the *SeT* transcript appeared to have a speckled cytoplasmic pattern, whereas its relative fluorescence levels were decreased in the cytoplasmic fraction of *miR-155*^*-/-*^ macrophages as compared to wild type cells (**Fig 5E**).

Altogether the aforementioned results lead to the conclusion that *miR-155* does not directly target the nascent *SeT* transcript in mouse macrophages upon proinflammatory activation with LPS. Additionally, deletion of *miR-155* can result in cytoplasmic *SeT* steady state RNA level reduction but, does not significantly alter nuclear *SeT* RNA levels, suggesting a synergistic-stabilizing relationship between *miR-155* and *SeT* RNA in macrophages.

## Discussion

Taken together, our data revealed that the non-coding genome either in the form of lncRNAs or microRNAs may play an active role in the regulation of the innate immune response via the regulation of chromatin organization and expression of the proinflammatory gene *Tnfα*, in murine macrophages. The allelic expression profile of a gene can quantitatively affect its overall mRNA levels, whereas lncRNA transcripts can participate in mechanisms affecting gene dosage control [62–64]. Allele biased expression is common amongst gene effectors of the immune system, as previously described for the monoallelically expressed *IfngR1* and *IfngR2* genes in lymphoid cells and the biallelically expressed *Tnfα* and *Arrb1* genes in LPS-stimulated murine macrophages [54,65]. Additionally, mechanisms favoring an allelic switch in response to external stimuli were implicated in heterogeneity promotion among individual cells within a broader population [66]. In this study we report that two players of the non-coding genome, namely the lncRNA *SeT* and *miR-155*, can modulate the allelic expression profile of the *Tnfα* gene. Utilizing genetically modified mice we showed that deletion of the regulatory elements of the lncRNA *SeT* resulted in the decrease of nascent *SeT* transcripts and in the upregulation of the *Tnfα* gene transcription. On the contrary, deletion of *miR-155*, unlike lncRNA *SeT*, resulted in the decrease of the *Tnfα* steady state mRNA levels and quite importantly in a reduced frequency of *Tnfα* expressing macrophage cells. Our findings are in accordance with what has previously been reported for the functional impact of non-coding genome mediators on gene regulation and regulation of epigenetic events during developmental and disease processes [67,68].

LncRNAs’ length and sequence specificity are suited to facilitate their allele-specific guiding role *in cis* or *in trans*, tethering protein factors to specific loci and affecting the expression of the latter. For instance *Xist*, *HOTAIR* and *ANRIL* lncRNAs exhibit a scaffolding role by targeting members of the Polycomb Repressive Complexes (PRC1 and PRC2), epigenetic factors and DNA methyltransferases [69,70]. In the case of the *cis*-acting *HOTTIP* RNA, it was shown that the transcript can act as a bridge between the MLL complex and the promoters at the *HOXA* cluster [71,72], while *NRON* ncRNA regulates the nuclear trafficking of NFAT [73]. *NEAT1* (*MEN3/b* in mouse), on the other hand, is an abundant, polyadenylated ncRNA that is an integral component of nuclear paraspeckles, which besides acting as a structural element, seems to also govern the nuclear export of mRNAs [74,75].

Apart from lncRNAs, miRNAs can also regulate proinflammatory gene expression in response to infection and efficient cessation of the former after pathogen eradication. As far as macrophage immune responses are concerned, our results regarding *miR-155* positively regulating *Tnfα* expression in primary murine macrophages are in line with previous reports accentuating its proinflammatory role. *MiR-155* has primarily been implicated in the initiation of the macrophage inflammatory response. Activation of TLRs by pathogens can initiate the MyD88-mediated signal transduction pathway leading NF-κB, MAPKs and members of the IRF family of transcription factors to activate macrophage pro-inflammatory genes [76]. *miR-155* was found upregulated after TLR4 exposure to LPS and was shown to directly target the *PU*.*1* [77] and *C/EBPβ* mRNAs after bacterial infection [78]. *MiR-155* was also shown to mediate host antiviral responses by repressing the suppressor of cytokine signaling 1 (SOCS1) expression, thus facilitating macrophage type I Interferon gene expression [33]. Additionally *miR-155* was found to positively affect *Tnfα* mRNA levels either by stabilizing the transcript or by targeting anti-inflammatory mediators of the innate response through an exosome-mediated transport [39,79].

The LPS response in mouse macrophages has been analyzed on a number of different approaches and such profiling revealed a cascade of gene regulation and several LPS-induced genes. However, it has not yet been possible to provide a reliable detailed map of the underlying regulatory transcriptional architecture. Our study revealed that the absence of lncRNA *SeT* and *miR-155* modulated *Tnfα* gene expression by formerly affecting the homologous pairing of *Tnfα* alleles. We previously showed that homologous pairing precedes the switch from mono- to bi-allelic expression of the *Tnfα* gene. Therefore regulation of *Tnfα* expression levels by these non-coding genome mediators may be mediated by higher order chromatin structure regulation of the *Tnfα* gene. The pairing of homologous chromosomes is a process that all eukaryotes perform at meiosis. In most organisms, obvious pairing is restricted to pre-meiotic germ cells, except for dipteran insects, in which somatic pairing is prominent in numerous cell types [80]. In addition, somatic pairing underlies several intriguing genetic and epigenetic phenomena involving both allelic and non-allelic interactions. There is evidence for low, but significant, levels of somatic homologous pairing in mice but also in humans. Cytological studies have revealed centromeric pairing of human chromosomes 1 and 17 in brain tissues [81] as well as homologous pairing of the pericentric regions in human lymphocytes [82]. Of particular interest are reports of chromosomal proximity in the region associated with the imprinted Prader-Willi and Angelman syndromes, the latter of which may be, in some cases, phenotypically and genetically related to autism and Rett Syndrome [83,84]. Homologous association of 15q11–13 domains has been observed during late S-phase in lymphocytes as well as in neurons. In mammals, the X-chromosome is unique in being capable of complete inactivation. The X-chromosome inactivation (XCI) process is initiated with counting the number of X chromosomes present in a nucleus, and initiation of XCI follows if this number exceeds one per diploid genome [85,86]. In order to ensure mutually exclusive silencing, the two X chromosomes are also regulated *in trans*. Interchromosomal pairing mediates this control [87], which occurs transiently at the onset of X inactivation (sensing) and is specific to the X-inactivation center (Xic) [88].

Our study indicated that homologous pairing of single gene alleles with vital importance can be regulated in multiple levels by the interplay of non-coding genome effectors. We found that upon deletion of the lncRNA *SeT* a vast increase in the biallelic expression profile of the *Tnfα* gene is observed. The nuclear function of lncRNA *SeT* could therefore be the coating of one of the two *Tnfα* alleles and the subsequent recruitment of negative regulators of transcription, early upon LPS stimulation, as a mode of biallelic gene transcription regulation. On the other hand *miR-155* is necessary for biallelic *Tnfα* gene transcription since upon deletion of the mature miRNA biallelic expression is diminished. Although we found that the mature *miR-155* does not directly target lncRNA *SeT* it seems to positively impact the stabilization of *SeT* transcript in the cytoplasm. on the contrary, in the absence of *SeT* lncRNA, *miR-155* can still convey its positive action as a constituent of a regulatory mechanism affecting both the homologous pairing and biallelic expression of *Tnfα* gene in the macrophage nucleus.

In conclusion, in this study we utilized genetically modified mice and we showed that lncRNA *SeT* and *miR-155* mediate *Tnfα* gene dosage control by regulating the homologous pairing of the two gene alleles and ultimately regulating its biallelic pattern. We suggest that this mechanism of homologous pairing regulating the allelic expression profile of *Tnfα* gene is of vital importance for the survival of innate immune cells after recurrent inflammatory stimulation and is thus controlled in multiple levels, including non-coding genome mediators. We further suggest that non-coding genome mediators might work as regulators of this higher order chromatin structure. Given that both lncRNAs and miRNAs can be encapsulated and secreted in exosomes [79,89], affecting primary responses in a systemic level, the elucidation of the mechanisms in which these mediators are involved can be implemented in disease-resolution approaches.
